# Comparing Multiple Linear Regression and Machine Learning in Predicting Diabetic Urine Albumin–Creatinine Ratio in a 4-Year Follow-Up Study

**DOI:** 10.3390/jcm11133661

**Published:** 2022-06-24

**Authors:** Li-Ying Huang, Fang-Yu Chen, Mao-Jhen Jhou, Chun-Heng Kuo, Chung-Ze Wu, Chieh-Hua Lu, Yen-Lin Chen, Dee Pei, Yu-Fang Cheng, Chi-Jie Lu

**Affiliations:** 1 Division of Endocrinology and Metabolism, Department of Internal Medicine, Department of Medical Education, Fu Jen Catholic University Hospital, School of Medicine, College of Medicine, Fu Jen Catholic University, New Taipei City 24352, Taiwan; liyinghuang@yahoo.com (L.-Y.H.); julia0770@yahoo.com.tw (F.-Y.C.); cpp0103@gmail.com (C.-H.K.); peidee@gmail.com (D.P.); 2 Graduate Institute of Business Administration, Fu Jen Catholic University, New Taipei City 242062, Taiwan; aaa73160@gmail.com; 3 Division of Endocrinology, Department of Internal Medicine, Shuang Ho Hospital, New Taipei City 23561, Taiwan; chungze@yahoo.com.tw; 4 Division of Endocrinology and Metabolism, Department of Internal Medicine, School of Medicine, College of Medicine, Taipei Medical University, Taipei 11031, Taiwan; 5 Division of Endocrinology and Metabolism, Department of Internal Medicine, Tri-Service General Hospital, School of Medicine, National Defense Medical Center, Taipei 11490, Taiwan; undeca2001@gmail.com; 6 Department of Pathology, Tri-Service General Hospital, National Defense Medical Center, Taipei 11490, Taiwan; anthonypatho@gmail.com; 7 Department of Endocrinology and Metabolism, Changhua Christian Hospital, Changhua 50051, Taiwan; cch143989@gmail.com; 8 Artificial Intelligence Development Center, Fu Jen Catholic University, New Taipei City 242062, Taiwan; 9 Department of Information Management, Fu Jen Catholic University, New Taipei City 242062, Taiwan

**Keywords:** type 2 diabetes, nephropathy, urine albumin-creatinine ratio, machine learning

## Abstract

The urine albumin–creatinine ratio (uACR) is a warning for the deterioration of renal function in type 2 diabetes (T2D). The early detection of ACR has become an important issue. Multiple linear regression (MLR) has traditionally been used to explore the relationships between risk factors and endpoints. Recently, machine learning (ML) methods have been widely applied in medicine. In the present study, four ML methods were used to predict the uACR in a T2D cohort. We hypothesized that (1) ML outperforms traditional MLR and (2) different ranks of the importance of the risk factors will be obtained. A total of 1147 patients with T2D were followed up for four years. MLR, classification and regression tree, random forest, stochastic gradient boosting, and eXtreme gradient boosting methods were used. Our findings show that the prediction errors of the ML methods are smaller than those of MLR, which indicates that ML is more accurate. The first six most important factors were baseline creatinine level, systolic and diastolic blood pressure, glycated hemoglobin, and fasting plasma glucose. In conclusion, ML might be more accurate in predicting uACR in a T2D cohort than the traditional MLR, and the baseline creatinine level is the most important predictor, which is followed by systolic and diastolic blood pressure, glycated hemoglobin, and fasting plasma glucose in Chinese patients with T2D.

## 1. Introduction

Type 2 diabetes (T2D) has become a growing global issue in recent decades. According to the 2021 Atlas of the International Diabetes Federation, it is estimated that there are 5.37 billion patients worldwide, and this trend will further increase to 6.0 billion by 2045 [1]. Not surprisingly, a similar endemic was noted in Taiwan. According to the data bank of the National Health Insurance Company, the total number of diabetic patients increased from 1.32 million to 2.2 million within 10 years (2005 to 2014). This represents an astonishing 66% increase [2]. It is now the 5th highest cause of death. In 2020, the cost spent on T2D was over 10 billion USD, which is approximately 4.66% of the budget of the National Health Insurance Company in one year. The accompanying complications, such as micro- and macrovascular diseases, impose heavy burdens on individuals and their families, as well as health providers and society [3,4]. It is important to note that this trend is particularly prominent among people aged <40 and ≥80 years [5]. 

Among all the complications, diabetic nephropathy is the leading cause of chronic kidney disease and end-stage renal disease (ESRD) [6], which are associated with high morbidity and mortality rate. According to the annual report of the US Renal Data System, Taiwan has the highest incidence (523 per million population) and prevalence of treated ESRD requiring renal replacement therapy [7]. In 2019, there were 84,615 dialysis patients and the National Health Insurance spent 1.54 billion, which is approximately 8.7–9.3% of the annual budget [8,9]. Therefore, its early detection and prevention are urgently required.

It is well known that urine albumin–creatinine ratio (uACR) is a strong predictor of the subsequent decline of the glomerular filtration rate in T2D, with an average of 0.93 mL per minute per month in approximately 35% of the subjects [10]. The underlying pathophysiology is due to the increased glomerular pressure, which is independent of hyperfiltration or hyperglycemia [11,12,13].

Traditionally, most studies have used multiple linear regression (MLR) to explore the relationships between risk factors and outcomes (complications) in medical research. Nevertheless, artificial intelligence using machine learning (ML), which enables machines to learn from past data or experiences without being explicitly programmed, has now become a new modality for data analysis that is competitive with MLR [14,15,16]. Because ML can capture nonlinear relationships in data and complex interactions among multiple predictors, it has the potential to outperform conventional MLR in disease prediction [17]. 

To our knowledge, only one study has attempted to predict the uACR in a T2D cohort. Thus, in the present study, we applied four different ML methods and attempted to answer the following questions in a diabetic cohort that was followed up for four years.

1. Compare the prediction accuracy between ML and traditional MLR.
2. Rank the importance of risk factors, such as demographic and biochemistry data.

## 2. Methods

### 2.1. Participant and Study Design

**Figure 1:**
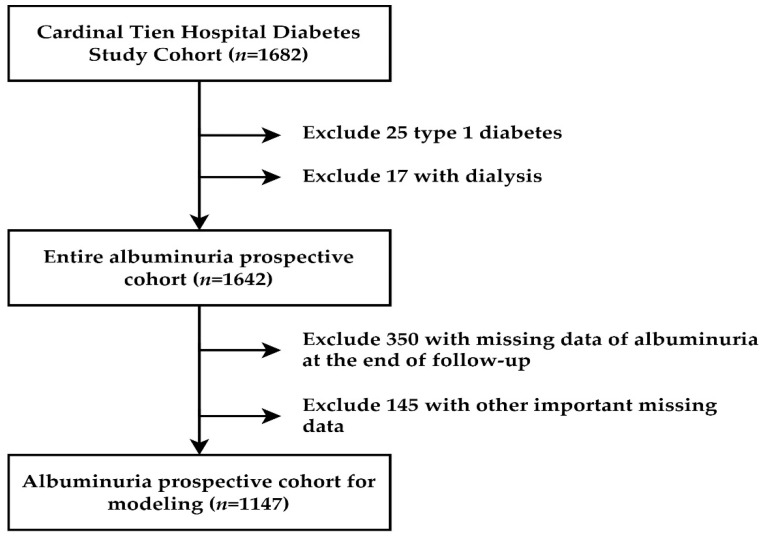
Flowchart of sample selection from the Cardinal Tien Hospital Diabetes Study Cohort.

Data for this study were obtained from the diabetic outpatient clinic of the Cardinal Tien Hospital in Taiwan from 2013 to 2019. This study is a prospective study, as we have collected our patients from 2013 to 2016. We designated this cohort as the Cardinal Tien Diabetes Study Cohort. Informed consent was obtained from all participants, and data were collected anonymously. The study protocol was approved by the Institutional Review Board of the hospital. In total, 1682 T2D patients were enrolled. After excluding subjects with different causes, 1147 subjects remained for analysis (women: 608, men: 539), as shown in Figure 1. They were followed up for 4 years. The following were the criteria for inclusion: (1) type 2 diabetes; (2) age between 50 and 75 years; (3) body mass in the range of 22–30 kg/m^2^; (4) glycated hemoglobin level between 6.5 and 10.5%; (5) the patients did not undergo regular dialysis. A flowchart of participant selection is displayed in Figure 1. 

On the day of the study, senior nursing staff recorded the subject’s medical history, including information on any current medications, and a physical examination was performed. The waist circumference was measured horizontally at the level of the natural waist. The body mass index (BMI) was calculated as the participant’s body weight (kg) divided by the square of the participant’s height (m). The systolic blood pressure (SBP) and diastolic blood pressure (DBP) were measured using standard mercury sphygmomanometers on the right arm of each subject while seated. 

As previously published, the procedures for collecting demographic and biochemical data are as follows [18]. After fasting for 10 h, blood samples were collected for biochemical analyses. Plasma was separated from the blood within 1 h of collection and stored at 30 °C until the analysis of fasting plasma glucose (FPG) and lipid profiles. FPG was measured using the glucose oxidase method (YSI 203 glucose analyzer; Yellow Springs Instruments, Yellow Springs, OH, USA). The total cholesterol and triglyceride (TG) levels were measured using the dry multilayer analytical slide method with a Fuji Dri-Chem 3000 analyzer (Fuji Photo Film, Tokyo, Japan). The serum high-density lipoprotein cholesterol (HDL-C) and low-density lipoprotein cholesterol (LDL-C) concentrations were analyzed using an enzymatic cholesterol assay, following dextran sulfate precipitation. A Beckman Coulter AU 5800 biochemical analyzer was used to determine the urine ACR by turbidimetry.

Table 1 lists the definitions of the 15 baseline clinical variables (independent variables, sex, age, BMI, duration of diabetes, smoking, alcohol use, FPG, glycated hemoglobin, triglyceride, HDL-C, LDL-C, alanine aminotransferase, creatinine (Cr), SBP, and DBP) used in this study. The uACR at the end of the follow-up was a numerical variable, which was used as a dependent (target) variable, while the remaining 15 variables were used as predictor variables in this study.

### 2.2. Proposed Scheme

This research proposed a scheme based on four machine learning methods, namely classification and regression tree (CART), random forest (RF), stochastic gradient boosting (SGB), and eXtreme gradient boosting (XGBoost), to construct predictive models for predicting diabetic uACR and to identify the importance of these risk factors. These ML methods have been applied in various healthcare applications and do not have prior assumptions regarding data distribution [19,20,21,22,23,24,25,26,27,28]. MLR was used as the benchmark for comparison.

The first method, CART, is a tree-structure method [29]. It is composed of root nodes, branches, and leaf nodes that grow recursively based on the tree structures from the root nodes and split at each node based on the Gini index to produce branches and leaf nodes with the rule. Then, the pruning node in the overgrown tree for optimal tree size using the cost-complexity criterion generates different decision rules to compose a complete structure tree [30,31].

RF, the second method in this study, is an ensemble learning decision tree algorithm that combines bootstrap resampling and bagging [32]. RF’s principle entails randomly generating many different and unpruned CART decision trees, in which the decrease in Gini impurity is regarded as the splitting criterion, and all generated trees are combined into a forest. Then, all the trees in the forest are averaged or voted to generate output probabilities and a final model that generates a robust model [33].

The third method, SGB, is a tree-based gradient boosting learning algorithm that combines both bagging and boosting techniques to minimize the loss function to solve the overfitting problem of traditional decision trees [34,35]. In SGB, many stochastic weak learners of trees are sequentially generated through multiple iterations, in which each tree concentrates on correcting or explaining errors of the tree generated in the previous iteration, that is, the residual of the previous iteration tree is used as the input for the newly generated tree. This iterative process is repeated until the convergence condition or a stopping criterion is reached for the maximum number of iterations. Finally, the cumulative results of many trees are used to determine the final robust model.

XGBoost, the fourth method of this study, is a gradient boosting technology based on an SGB optimized extension [36]. Its principle is to train many weak models sequentially to ensemble them using the gradient boosting method of outputs, which achieves a better prediction performance. In XGBoost, Taylor binomial expansion is used to approximate the objective function and arbitrary differentiable loss functions to accelerate the model construction convergence process [37]. Then, XGBoost applies a regularized boosting technique to penalize the complexity of the model and correct overfitting, thus increasing model accuracy [36].

**Figure 2:**
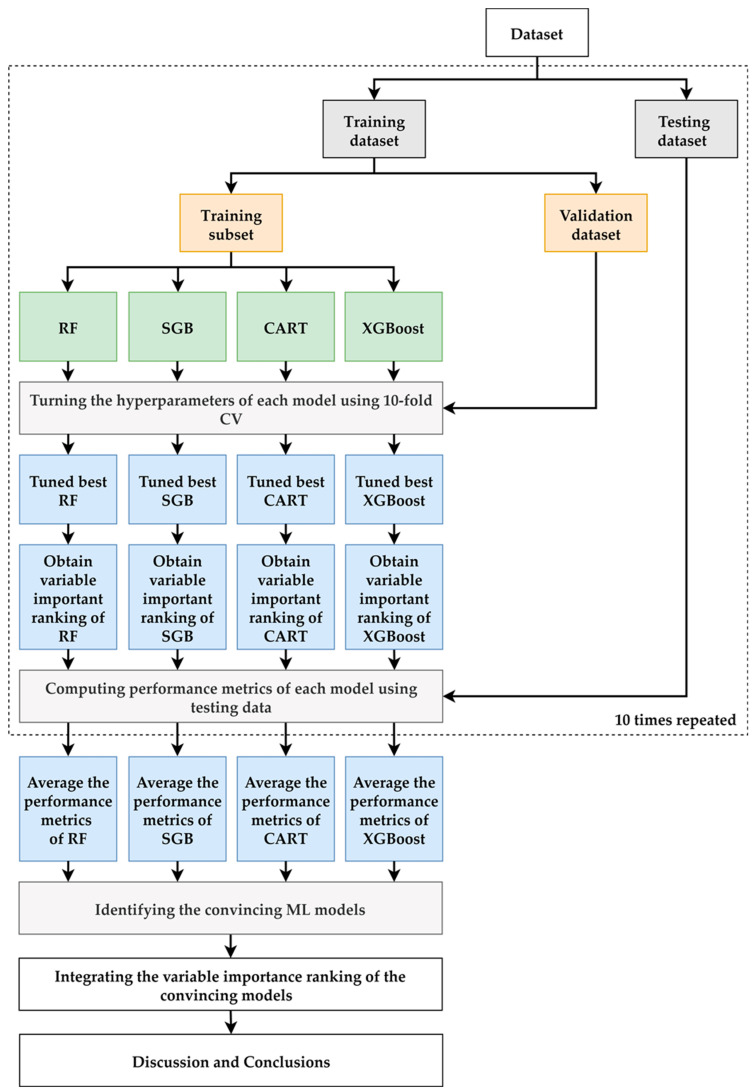
Proposed ML prediction scheme.

A flowchart of the proposed prediction and important variable identification scheme that combines the four ML methods is shown in Figure 2. First, patient data were collected using the proposed method to prepare the dataset. The dataset was then randomly divided into an 80% training dataset for model building and a 20% testing dataset for model testing. In the training process, each ML method has its hyperparameters that must be tuned to construct a relatively well-performed model. In this study, a 10-fold cross-validation (CV) technique for hyperparameter tuning was used. The training dataset was further randomly divided into a training dataset to build the model with a different set of hyperparameters and a validation dataset for model validation. All possible combinations of the hyperparameters were investigated using a grid search. The model with the lowest root mean square error for the validation dataset was viewed as the best model for each ML method. The best turned RF, SGB, CART, and XGBoost models were generated, and the corresponding variable importance ranking information was obtained.

During the testing process, the testing dataset was used to evaluate the predictive performance of the best RF, SGB, CART, and XGBoost models. As the target variable of the models built in this study is a numerical variable, the metrics used for model performance comparison are the mean absolute percentage error (MAPE), symmetric MAPE (SMAPE), and relative absolute error (RAE), which are shown in Table 2. 

To provide a more robust comparison, the training and testing processes mentioned above were randomly repeated 10 times. The averaged metrics of the RF, SGB, CART, and XGBoost models were used to compare the model performance of the benchmark MLR model that used the same training and testing dataset as the ML methods. An ML model with an average metric lower than that of MLR was considered a convincing model.

Because all of the ML methods used can produce the importance ranking of each predictor variable, we defined that the priority demonstrated in each model ranked 1 as the most critical risk factor and 15 as the last selected risk factor. The different ML methods may produce different variable importance rankings because they have different modeling characteristics; therefore, we integrated the variable importance ranking of the convincing ML models to enhance the stability and integrity of re-ranking the importance of risk factors. In the final stage of the proposed scheme, we summarize and discuss our significant findings regarding the convincing ML models and identify important variables.

In this study, all methods were performed using R software version 4.0.5 and RStudio version 1.1.453 with the required packages installed (http://www.R-project.org, accessed on 1 February 2022; https://www.rstudio.com/products/rstudio/, accessed on 1 February 2022). The implementations of RF, SGB, CART, and XGBoost were the “randomForest” R package version 4.6-14 [38], “gbm” R package version 2.1.8 [39], “rpart” R package version 4.1-15 [40], and “XGBoost” R package version 1.5.0.2, respectively [41]. In addition, to estimate the best hyperparameter set for the developed effective CART, RF, SGB, and XGBoost methods, the “caret” R package version 6.0–90 was used [42]. The MLR was implemented using the “stats” R package version 4.0.5, and the default setting was used to construct the models.

## 3. Results

A total of 1147 participants were enrolled in the study (men: 539, women: 608). The demographic data are shown in Table 3 (mean ± standard deviation). The results of the comparison between the traditional MLR and the four ML methods (i.e., RF, SGB, CART, and XGBoost) in predicting diabetic uACR in a 4-year follow-up cohort are shown in Table 4. From the table, it can be seen that all four ML methods yielded lower prediction errors than the MLR method and were all convincing ML models. To determine whether the four ML methods significantly outperformed the MLR method, the Wilcoxon signed-rank test was used. The Wilcoxon signed-rank test is one of the most popular distribution-free, non-parametric statistical tests for evaluating the performance of two prediction models [43]. Table 5 shows the test results of the four ML methods and the MLR method. It can be observed from the table that the prediction error values of all ML methods were significantly different from those of the MLR method. Therefore, it can be determined that the ML methods used in this study significantly outperformed traditional MLR in predicting uACR at the end of the follow-up in terms of prediction error.

Table 6 presents the average importance ranking of each factor generated by the RF, SGB, CART, and XGBoost methods. It can be observed from the figure that the different ML methods generated different relative importance rankings for each factor. The darkness of the blue color indicates the importance of risk factors. The darker the blue color, the more important the risk factor. For instance, in the RF method, the first three important factors were baseline Cr, age, and baseline SBP. The most important feature of the SGB method was baseline Cr, which was followed by baseline HDL-C and baseline DBP. To fully integrate the importance rankings of each factor in all the four ML methods, the average importance ranking of each risk factor was obtained by averaging the ranking values of each variable in each method. 

**Figure 3:**
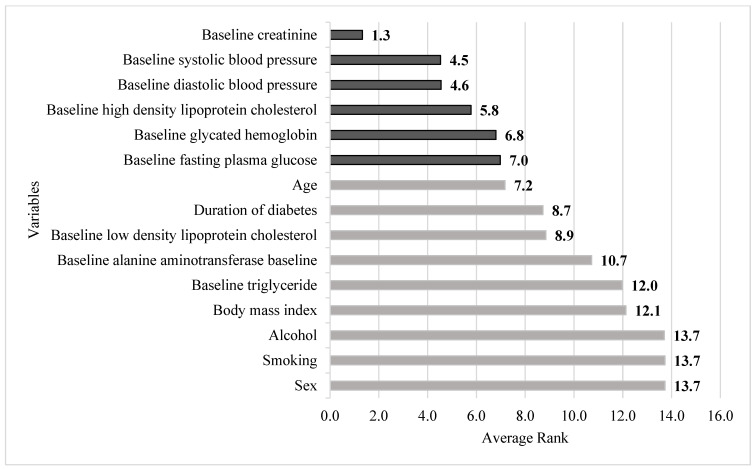
Integrated importance ranking of all risk factors. Note: The darker color indicates the first six important risk factors of this study.

Figure 3 depicts the risk factors based on the increasing order of the averaged ranking values. It can be noted from the figure that the first six important risk factors in predicting diabetic uACR in a 4-year follow-up cohort are baseline Cr, baseline SBP, baseline DBP, baseline HDL-C, baseline glycated hemoglobin, and baseline FPG.

## 4. Discussion

As mentioned in the Introduction, the present study has two goals. The first was to compare the accuracy between ML methods and MLR, and the second was to identify the rank of different risk factors for predicting uACR. Our study showed that all four ML methods outperformed the MLR. We also found that baseline Cr, blood pressure, HDL-C, glycated hemoglobin, and FPG were the most important factors.

Traditionally, MLR has been widely used to analyze medical research to deal with continuous variables. However, it is difficult to describe the nonlinear data patterns of MLR, and the effective use of MLR requires fitting its strong assumptions during modeling. Unlike MLR, ML does not require strong model assumptions and can capture the delicate underlying nonlinear relationships contained in empirical data [19]. Our present data showed that all four ML methods are superior to MLR because the MAPE and RAE of the ML methods all have lower values (Table 4). Our results suggest that ML might have a great potential for medical studies and applications.

Because diabetic nephropathy causes a serious burden on individuals and consumes a large portion of the government health budget, extensive studies have focused on this topic [6,44,45,46,47]. From these previous studies, it could be concluded that sex, high blood glucose and blood pressure, smoking, dyslipidemia, decreased glomerular filtration rate, BMI, and uACR are common risk factors for future uACR. However, in the present study, our data showed that baseline Cr, DBP, SBP, HDL-C, glycated hemoglobin, and FPG were the most important risks. Additionally, the roles of diabetes duration, glycated hemoglobin, BMI, HDL-cholesterol, triglyceride, sex, smoking, and alcohol use were less important. 

Our data suggest that the most important predictor of albuminuria is baseline Cr. This is not surprising because albuminuria occurs early in the course of diabetic nephropathy [48]. According to the majority of previous studies, a summary of this relationship could be depicted as follows: diabetic patients with albuminuria are at a higher risk of end-stage renal and cardiovascular diseases [49,50]. This indicates that albuminuria is the cause of end-stage renal disease, which differs from the findings of the present study. Our results show that an increase in serum Cr level could predict albuminuria four years later, which is an opposite cause–effect relationship to the majority of the other studies. However, our finding can be supported by the cornerstone study conducted by Gansevoort et al. [51]. This meta-analysis clearly showed that there are independent, continuous, and negative associations between serum Cr and albuminuria. Thus, it could be postulated that each of these factors could affect the other at the same time. Further research is required to explore this area.

Both diastolic and systolic blood pressures were identified as the second and third important factors for predicting albuminuria. Their relationships are well known and have been extensively studied [52]. Similar to the role of increased serum Cr levels, kidney disease causes an increase in BP, which could further deteriorate renal function. More specifically, the change in BP is in concordance with and even precedes albuminuria [53]. By controlling BP, the speed of end-stage renal disease progression can be slowed down [54]. 

Interestingly, HDL cholesterol level was the only lipid found to be correlated with albuminuria. However, few studies have focused on this topic. Most previous studies have demonstrated that different stages of diabetic kidney disease (DKD) have different influences on blood lipid levels [55,56]. Other studies measured apolipoproteins and the size of LDL-cholesterol, which all showed positive correlations with DKD, including albuminuria [57]. To our knowledge, only two studies are relatively close to the present findings. The first study was performed by Sacks et al. In a group of 2535 T2D patients, they evaluated the impact of HDL-C levels on uACR. Furthermore, kidney disease was defined as albuminuria, proteinuria, or decreased eGFR. The data showed that the odds ratio of having kidney disease decreased by 0.86 (0.82–0.91) for every 0.2 mmol/L (approximately 1 quintile) increase in HDL-C [58]. The second study was conducted on a cohort of 524 Chinese patients. Using multiple logistic regression, after adjusting for the available confounding factors, they suggested that subjects with the highest quartile HDL-C had a lower odds ratio (OR = 0.17, 95% confidence interval 0.15–0.52) of having uACR than the lowest quartile. However, a limitation of this study was that it was cross-sectional. Thus, it was unable to infer the causation or directionality of this relationship [59]. This study responds to this limitation in its longitudinal design. The causative influence of HDL-C level can be explained by several assumptions. First, the glomerular and renal tubules could be injured by impaired HDL-C function, which hinders the reversal of the cholesterol transport process [60]. Second, the antioxidative ability of the HDL-C is reduced and oxidative stress is increased, which further influences the immune-mediated diabetic nephropathy [61]. Finally, it is well known that low HDL-C levels are associated with insulin resistance, hyperinsulinemia, and hyperglycemia. All these untoward derangements can damage endothelial cells in the glomerulus [62,63].

The last two factors affecting albuminuria are glycated hemoglobin and FPG levels. This finding is compatible with the results of the Diabetes Control and Complication Trial (DCCT) [64]. The data showed positive relationships between glucose control and albuminuria. Moreover, after controlling for blood glucose levels, albuminuria also improved [65]. Because DCCT enrolled patients with type 1 diabetes, its pathophysiology is different from that of the present study. Regarding T2D, few studies have been conducted in this area. A comprehensive meta-analysis conducted by Lo et al. [66] showed that for intensive control (glycated hemoglobin < 7% and FPG < 6.6 mmol/L), the relative risk of having uACR was 0.59 (confidence interval: 0.38–0.93). As this study enrolled 11 studies (29,141 subjects) and follow-ups were conducted for an average of 56.7 months, their conclusion is convincing. The underlying pathophysiology to support this result is that high blood glucose concentration could involve mesangial cell damage in nephrons [67]. However, it is worth noting that both A1c and FPG were classified as important predictors. This might indicate that because FPG is only one blood glucose measurement within 90 days compared to A1c, it is less accurate than A1c. Our results show that they are ‘independent’ of each other.

Interestingly, in the present study, the duration of diabetes, body mass index, sex, smoking, and alcohol use were less important. This finding could be attributed to the nature of the ML. ML methods are data-driven, non-parametric models. They can map any nonlinear function without an a priori assumption about the properties of the data and have the ability to capture subtle functional relationships among the empirical data, even though the underlying relationships are unknown or difficult to describe [68,69,70]. These factors may contain richer linear pattern information and less important nonlinear information than baseline creatinine, blood pressure, albuminuria level, and age. Thus, they were ranked as less important risk factors using ML methods. 

This study had some limitations. First, the smoking and alcohol details need to be more defined because some other reports have shown that they have an important impact on the occurrence of diabetic nephropathy. Second, we did not collect information on the use of angiotensin-converting enzyme inhibitors, angiotensin receptor blockers, sodium-glucose cotransporter 2 inhibitors, and glucagon-like peptide-1 agonists. All these medications would have beneficial effects on DKD. Third, some of the data, such as uACR and blood pressure, were collected only once. For some of the participants, we did have data more than once. However, because the number is less than the present number, we still chose to enroll subjects with only one value. Even though these drawbacks do exist, our large n number and the characteristics of ML (alleviating the effects of extremes) could at least partially adjust.

## 5. Conclusions

ML might be more accurate in predicting uACR in T2D than the traditional MLR, and the baseline creatinine level is the most important factor to predict uACR in a T2D cohort, which is followed by systolic and diastolic blood pressure, glycated hemoglobin, and fasting plasma glucose.

## Figures and Tables

**Table 1:** Variable definition.

Variables	Description	Unit
Sex	Male/Female	-
Age	Patient age	year
Body mass index	Body mass index	Kg/m^2^
Duration of diabetes	Duration of diabetes	year
Smoking	No/Yes	-
Alcohol	No/Yes	-
Baseline fasting plasma glucose	Fasting plasma glucose baseline	mg/dL
Baseline glycated hemoglobin	HbA1c (Glycated hemoglobin) baseline	%
Baseline triglyceride	Triglyceride baseline	mg/dL
Baseline high-density lipoprotein cholesterol	High-density lipoprotein cholesterol baseline	mg/dL
Baseline low-density lipoprotein cholesterol	Low-density lipoprotein cholesterol baseline	mg/dL
Baseline alanine aminotransferase baseline	Alanine aminotransferase baseline	U/L
Baseline creatinine	Creatinine baseline	mg/dL
Baseline systolic blood pressure	Systolic blood pressure baseline	mmHg
Baseline diastolic blood pressure	Diastolic blood pressure baseline	mmHg
uACR at the end of follow-up	Urine albumin to creatinine ratio = albumin (mg/dL)/urine creatinine (mg/dL) follow up 4 year	mg/g

uACR: urine albumin–creatinine ratio.

**Table 2:** Equation of Performance Metrics.

Metrics	Description	Calculation
MAPE	Mean Absolute Percentage Error	$MAPE=\frac{1}{n}\overset{n}{\underset{i=1}{\sum }}\left|\frac{y_{i}-{\hat{y}}_{i}}{y_{i}}\right|\times 100$
SMAPE	Symmetric Mean Absolute Percentage Error	$SMAPE=\frac{1}{n}\overset{n}{\underset{i=1}{\sum }}\frac{\left|y_{i}-{\hat{y}}_{i}\right|}{\left(\left|y_{i}\right|+\left|{\hat{y}}_{i}\right|\right)/2}\times 100$
RAE	Relative Absolute Error	$RAE=\sqrt{\frac{\sum_{i=1}^{n}{\left(y_{i}-{\hat{y}}_{i}\right)}^{2}}{\sum_{i=1}^{n}{\left(y_{i}\right)}^{2}}}$

where ${\hat{y}}_{i}$ and $y_{i}$ represent predicted and actual values, respectively; n stands the number of instances.

**Table 3:** Participant demographics.

Variables	Mean ± SD	N
Age	63.82 ± 11.49	1123
BMI	26.45 ± 3.95	1134
Duration of diabetes	14.13 ± 7.65	1137
Baseline fasting plasma glucose	149.84 ± 42.80	1146
Baseline glycated hemoglobin	7.74 ± 1.49	1140
Baseline triglyceride	142.99 ± 94.55	1144
Baseline high-density lipoprotein cholesterol	44.87 ± 12.00	845
Baseline low-density lipoprotein cholesterol	98.82 ± 27.73	1129
Baseline alanine aminotransferase baseline	29.38 ± 21.48	1134
Baseline creatinine	0.90 ± 0.37	1093
Baseline systolic blood pressure	131.13 ± 14.07	969
Baseline diastolic blood pressure	75.91 ± 11.66	969
uACR at the end of follow-up	195.30 ± 711.98	1147
	**N (%)**	**N**
Sex		1147
Male	608 (53.01%)	
Female	539 (46.99%)	
Smoking		716
No	430 (60.06%)	
Yes	286 (39.94%)	
Alcohol		789
No	715 (90.62%)	
Yes	74 (9.38%)	

BMI: body mass index. uACR: urine albumin–creatinine ratio.

**Table 4:** The average performance of the MLR, RF, SGB, CART, and XGBoost methods.

	MAPE	SMAPE	RAE
MLR	18.245 (4.79)	1.545 (0.04)	1.126 (0.17)
RF	16.174 (4.82)	1.266 (0.05)	1.072 (0.19)
SGB	14.850 (3.09)	1.522 (0.07)	1.040 (0.16)
CART	9.528 (1.76)	1.312 (0.06)	0.841 (0.10)
XGBoost	11.872 (2.80)	1.274 (0.06)	0.915 (0.11)

MLR: multiple linear regression; RF: random forest; SGB: stochastic gradient boosting; CART: classification and regression tree; XGBoost: eXtreme gradient boosting; MAPE: mean absolute percentage error; SMAPE: symmetric mean absolute percentage error; RAE: relative absolute error.

**Table 5:** Wilcoxon sign-rank test between four ML methods and MLR method.

	RF	SGB	CART	XGBoost
MLR	41.736 (0.001) **	20.814 (0.001) **	30.680 (0.001) **	44.489 (0.001) **

The numbers in parentheses are the corresponding *p*-value; **: *p* < 0.05.

**Table 6:** Importance ranking of each risk factor using the four convincing methods.

Variables	RF	SGB	CART	XGBoost	Average	
Sex	11.3	14.9	15.0	13.7	13.7	
Age	4.8	9.0	9.5	5.4	7.2	
Body mass index	14.9	11.8	12.0	9.8	12.1	
Duration of diabetes	8.8	7.0	10.7	8.4	8.7	Rank value
Smoking	10.8	14.4	15.0	14.7	13.7	1.0~1.4
Alcohol	11.6	13.6	15.0	14.6	13.7	1.5~2.4
Baseline fasting plasma glucose	5.4	6.3	10.9	5.3	7.0	2.5~3.4
Baseline glycated hemoglobin	5.8	5.0	10.3	6.1	6.8	3.5~4.4
Baseline triglyceride	11.9	10.2	12.7	13.1	12.0	4.5~5.4
Baseline high-density lipoprotein cholesterol	7.7	2.8	5.8	6.8	5.8	5.5~
Baseline low-density lipoprotein cholesterol	5.8	10.9	11.2	7.5	8.9	
Baseline alanine aminotransferase baseline	9.6	8.3	12.4	12.6	10.7	
Baseline creatinine	1.3	1.1	1.8	1.1	1.3	
Baseline systolic blood pressure	5.0	4.9	4.3	3.9	4.5	
Baseline diastolic blood pressure	5.3	4.1	4.1	4.7	4.6	

Note: Different blue colors indicate different rank values of risk factors. The darker the blue color, the more important the risk factor.

## Data Availability

Data available on request due to privacy/ethical restrictions.
